# Abortion care pathways and service provision for adolescents in high-income countries: A qualitative synthesis of the evidence

**DOI:** 10.1371/journal.pone.0242015

**Published:** 2020-11-09

**Authors:** Anisa R. Assifi, Melissa Kang, Elizabeth A. Sullivan, Angela J. Dawson

**Affiliations:** 1 The Australian Centre for Public and Population Health Research, Faculty of Health, University of Technology Sydney, Sydney, Australia; 2 Office of the PVC Health and Medicine, Faculty of Health and Medicine, University of Newcastle, Callaghan, Australia; Aga Khan University Medical College Pakistan, PAKISTAN

## Abstract

Limited research in high-income countries (HICs) examines adolescent abortion care-seeking pathways. This review aims to examine the pathways and experiences of adolescents when seeking abortion care, and service delivery processes in provision of such care. We undertook a systematic search of the literature to identify relevant studies in HICs (2000–2020). A directed content analysis of qualitative and quantitative studies was conducted. Findings were organised to one or more of three domains of an a priori conceptual framework: context, components of abortion care and access pathway. Thirty-five studies were included. Themes classified to the Context domain included adolescent-specific and restrictive abortion legislation, mostly focused on the United States. Components of abortion care themes included confidentiality, comprehensive care, and abortion procedure. Access pathway themes included delays to access, abortion procedure information, decision-making, clinic operation and environments, and financial and transportation barriers. This review highlights issues affecting access to abortion that are particularly salient for adolescents, including additional legal barriers and challenges receiving care due to their age. Opportunities to enhance abortion access include removing legal barriers, provision of comprehensive care, enhancing the quality of information, and harnessing innovative delivery approaches offered by medical abortion.

## Introduction

Universal access to safe medical and surgical abortion is an essential element of comprehensive, high-quality adolescent reproductive healthcare [1]. Despite adolescent fertility falling in many high-income countries (HICs), unprotected sex, contraceptive failure, sexual violence, maternal illness, or fetal anomaly may lead to a need for access to safe abortion. The term HICs is used as defined by the World Bank classification which is based on Gross National Income (GNI) per capita calculated using the atlas method [2].

Abortion remains a relatively common outcome of adolescent pregnancy despite an overall downward trend in abortion incidence among adolescents in HICs [3]. A recent study examined complete adolescent abortion and pregnancy data and found that 17%-69% of pregnancies resulted in abortion [4]. And in half of the countries 35% - 55% of pregnancies ended in abortion. It is therefore important to examine the care-seeking pathways in order to improve access to abortion and experience of abortion for adolescents.

Adolescent females (< 20 years) encounter greater challenges and barriers when accessing abortion services and information compared to older women. The stigma associated with premarital sexual activity, unplanned pregnancy and abortion [5, 6] and a lack of reproductive health information can affect an adolescents’ knowledge of her pregnancy status and health-seeking behaviour which may delay her access to services. Abortion stigma is multifaceted: it is experienced, perceived, and internalized, often leading to a desire to keep a pregnancy or subsequent abortion secret [6].

Access to abortion for all age groups is affected by the availability of and distance to services, their acceptability to women, the cost of the procedure, and the legal context [1, 7]. Abortion legislation varies within and between countries. Even in countries with liberal abortion laws, restrictions may exist within the country around gestational age limits, and when and where a woman can legally obtain an abortion [1]. Adolescents may be subjected to additional restrictive laws, such as parental notification and consent laws, and judicial authorization.

Studies from low- and middle-income countries (LMICs) have identified factors affecting adolescent access to abortion. These include delays in care-seeking due to lack of knowledge affecting the recognition and acknowledgment of pregnancy, fear of disclosure, the cost of the procedure may increase the likelihood of adolescents presenting to services in the second trimester of pregnancy [8–11]. In addition, adolescents may seek the use of unskilled abortion providers, due to legal barriers and the fear of abortion provider judgment, that is associated with complications and adverse outcomes [9, 12, 13]. Discriminatory attitudes of staff can delay or prevent adolescents from receiving abortions, leaving them feeling mistreated [14]. Adolescents in HICs may encounter similar delays and experience stigma to those in LMIC. However they are less likely to access clandestine services as only 0.9% of abortions in HICs across all women are considered ‘least safe’ (involving untrained personnel using dangerous, invasive methods) [15]. Compared to low-income countries where 53.8% of abortion are considered ‘least safe’ [15]. There is limited research in HICs examining abortion care-seeking pathways, and even less that focuses on adolescents as the key population group. Two reviews by Doran et al. and Dawson et al. provide insight into abortion service delivery in HICs however, the needs of adolescents were not explicitly investigated [16, 17].

We undertook a directed content analysis of the evidence to address the lack of knowledge concerning how adolescents’ access, navigate and experience abortion services as part of broader sexual and reproductive health care in HICs. The processes involved in making a decision to have an abortion were included in this review as it is considered the beginning and entry point of adolescent’s abortion care-seeking pathway. This review aims to examine the pathways and experiences of adolescents when seeking abortion care, and service delivery processes in provision of care.

## Methods

### Protocol registration

We registered this review with Prospero [CRD42018067256] [18].

### Search strategy

We searched for relevant primary research published in peer-reviewed journals from 2000 to the present, using Google Scholar and the databases: PubMed, Medline, Embase, CINAL, Scopus, Informit and Popline (S1 Table). We manually searched the reference lists of included articles and relevant journals (e.g., International Journal of Adolescence and Youth, etc.).

Controlled vocabulary keywords (MeSH) and free-text words included “adolescent” OR “abortion” OR “service delivery” were employed to search databases (Table 1).

**Table 1:** Additional search terms.

Key terms	Alternative search terms
Adolescent	adolescents, teen, teens, teenager, youth, youths, adolescent girls, young people
Abortion	abortions, termination of pregnancy, pregnancy termination, induced abortion, legal abortion, menstrual regulation
Service delivery	health service, abortion service, adolescent friendly service, youth friendly service, model of care

**Fig 1:**
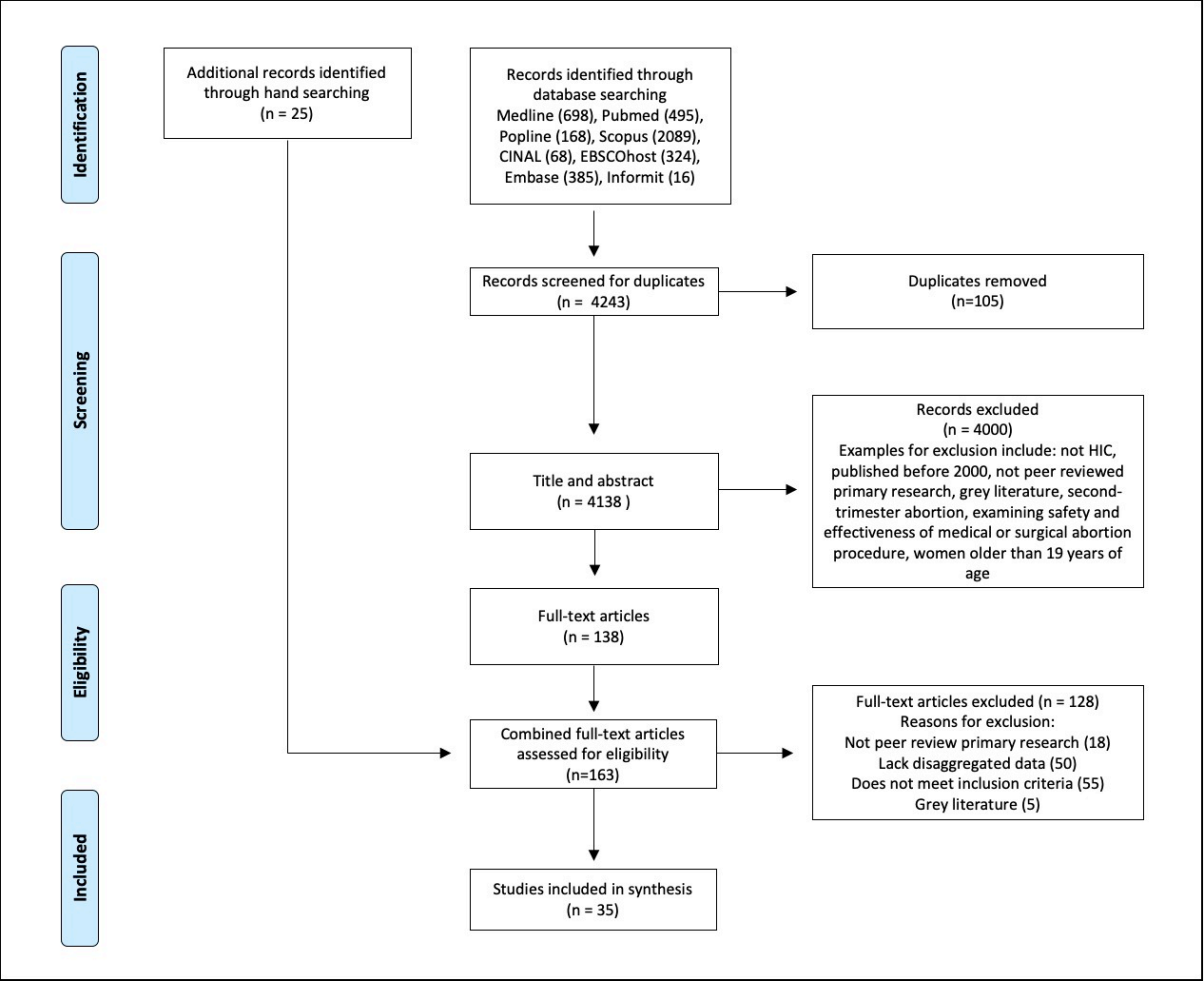
PRISMA diagram.

Table 2 provides the inclusion and exclusion criteria. The PRISMA diagram (Fig 1) outlines the information flow through the systematic search. The search yielded 4,243 citations, of these 138 full text articles were read and discussed by authors AA, MK and AD. Twenty-seven studies were included for analysis. Handsearching of reference lists by AA and AD identified a further eight studies, bringing the total number of included studies to 35.

**Table 2:** Study inclusion and exclusion criteria.

Included	Excluded
Original/primary research article.	Discursive/descriptive, incidence, prevalence, grey literature studies etc.
Sample includes adolescent girls aged 10 to 19 years of age (defined by WHO [19]).	Women >19 years of age.
High-income country (defined by World Bank [2]).	Low- and middle-income country.
Research examining first trimester abortion service delivery.*	Research examining second trimester abortion service delivery.
	Research examining the safety and effectiveness of medical and surgical abortion procedures.
Research examining adolescents’ abortion decision-making.	
Induced abortion service provision (medical and surgical methods).	Miscarriage, threatened abortion, missed abortion.
Research examining adolescents and provider’s experience and perception of the abortion service delivery pathway.	Research examining adolescent’s and provider’s views on abortion and whether adolescents should have access.
Research examining adolescent’s and provider’s experience at abortion counselling pre- and post-abortion.	
English	Non-English
Articles published since 2000.	Articles published before the year 2000.

*First trimester abortions were included in this review as the majority of abortions amongst adolescents in HIC occur in the first trimester [3]. Second and later trimester abortions were excluded to understand the nature of common adolescent experiences.

### Data extraction

Data were extracted according to 1) publication characteristics (e.g., authors, year of publication), 2) study design, setting and methodology, 3) characteristics of the study population (e.g., age, socio-demographic data), and 4) study findings (e.g., abortion service model of care, study population’s experience and perception of the service delivery). Of the 35 studies, seven of the studies’ authors were contacted to ask about disaggregation of relevant data by age. One author responded [20, 21].

### Quality assessment

We used the Critical Appraisal Skills Programme checklist to appraise qualitative studies [22]. Quantitative studies were assessed using a variation of the Newcastle-Ottawa Scale (NOS) assessment tools [23] and the Mixed Methods Appraisal Tool was used to assess mixed-method studies [24]. Quality assessment was undertaken by one author (AA) and a second author (AD) independently verified. Differences were discussed between authors AA and AD, and agreement reached. No studies were excluded. See S2 Table.

### Data analysis

**Fig 2:**
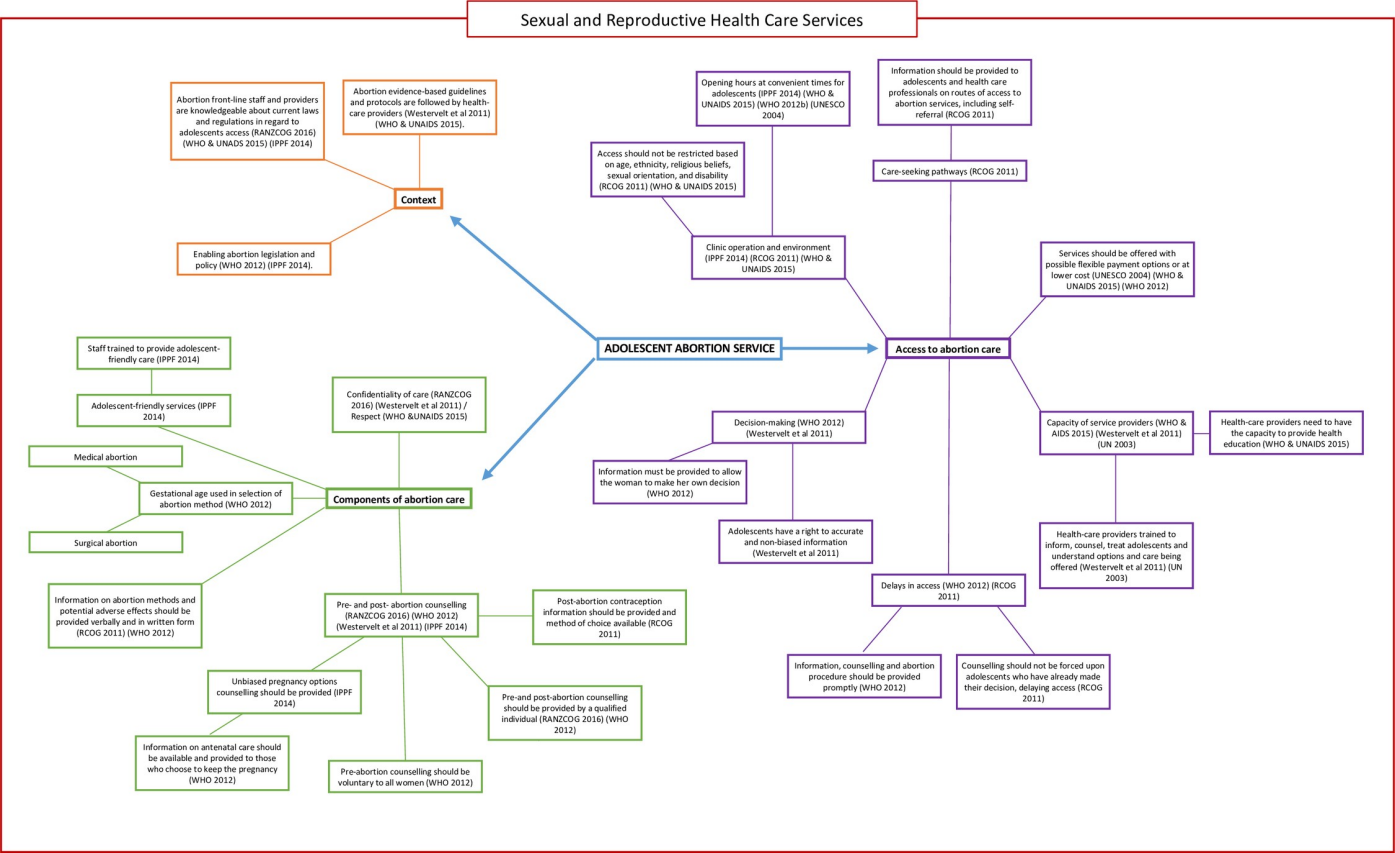
A priori framework for adolescent abortion service delivery.

We undertook a directed content analysis of the findings of each paper, as per the steps outlined in Hsieh and Shannon 2005 [25]. An *a priori* framework was used to identify the key elements that underpin the delivery of abortion services for adolescents (Fig 2). We first undertook a scoping review to identify existing national and international abortion care guidelines that included adolescent-specific care and adolescent-friendly health service guidelines. These findings informed the conceptualisation of the framework. Recommendations from the guidelines were categorised into three domains: (i) context, (ii) components of abortion care, and (iii) access pathways to abortions for young women. ‘Context’ is concerned with the policy environment of abortion service delivery, including legislation, availability of evidence-based guidelines and service provider awareness of both. ‘Components of abortion care’ comprises recommendations on the delivery of abortion services, e.g., confidentiality of care, gestational age assisting in method choice, counselling, and adolescent-friendly service. ‘Access pathways to abortion’ is concerned with how adolescents identify information and access an abortion service.

The principal author read each study and developed a set of codes informed by the framework. Codes were organised into categories and sub-categories and then themes that were organised to the three domains of the a priori framework. The categories and sub-categories were reviewed and validated by the authors. Themes and sub-themes were finalised following a second round of coding across all studies.

## Results

Thirty-five studies were included in the review and are summarised in S3 Table. Studies were published from 2001 to 2019, with 12 published in the last five years [20, 26–36]. Sixteen studies were cross-sectional quantitative [20, 26, 29–31, 35–45], 13 were cross-sectional qualitative [27, 33, 34, 46–55], four were mixed method [56–59], and two were quantitative cohort studies [28, 32]. The study data covered eight countries (Australia, Israel, Netherlands, New Zealand, Portugal, Sweden, UK and the United States), most from the United States (US) [20, 26–28, 30–32, 34, 37–39, 41, 42, 44–46, 48–50, 54, 56–59].

Table 3 summarises the findings according to the three overarching domains of the framework (context, components of abortion care, and access pathways to abortions for young women) and their associated themes and sub-themes.

**Table 3:** Results: Content analysis of study findings.

Overarching domains	Themes	Sub-themes	Key topics areas	Country of study	References
Context	Abortion legislation	Restrictive legislation	Effects due to general restrictive abortion legislation	US	[27]
			Gestational age limit	US	[37, 44]
		Adolescent specific legislation	Parental notification law	Netherlands, Sweden, UK, US	[28, 30, 31, 37, 38, 54]
			Judicial bypass	US	[28, 34, 37, 48, 53, 57]
Components of abortion care	Confidentiality			US	[46, 48, 57]
	Comprehensive care			Israel Netherlands, Sweden, UK, US	[35, 36, 47, 53–55]
	Abortion procedure			Sweden, US	[41, 49, 51]
Access to abortion care	Delays in access	Individual delays		UK, US	[20, 53, 58]
		System delays		New Zealand, US	[43, 58]
		Effects of delays		US	[20, 45]
	Information about abortion procedure			Netherlands, Sweden, UK, US	[33, 46, 48, 49, 54]
	Decision-making process			Australia, Netherlands, Portugal, Sweden, UK, US	[29, 33, 42, 46, 47, 51–54, 56–59]
	Clinic operation and environment	Effect of protestors		US	[39]
		Telemedicine		US	[50]
	Care-seeking pathway	Structural barriers		Australia, Netherlands, Sweden, UK, US	[20, 26, 27, 32, 37, 40, 45, 46, 50, 54]

### Context

Twelve studies reported findings that were mapped to the domain ‘Context’. All themes and sub-themes were related to abortion legislation and judicial processes.

#### Abortion legislation

‘Restrictive abortion legislation’ created greater challenges for adolescents to access services whilst trying to maintain confidentiality from their family. Fuentes et al. reported women’s experiences accessing abortion in Texas after the 2013 House Bill 2 (HB2) was passed. A Bill which further restricted abortion service provision resulting in the closures of abortion facilities [27]. Adolescents described trying to work out how to keep their pregnancy and subsequent abortion a secret from their parents [27]. Adolescents stated that legislative changes affected their ability to secure an abortion privately as availability of services had reduced. As a result adolescents had to seek assistance from their parents to access a clinic [27]. In 2016 HB2 was overturned by the US Supreme Court [60, 61].

Under the ‘Clinic-based gestational age limits’ theme, in a five-year longitudinal study 35% of adolescents 15 to 17 were turned away from services as they were over the clinic’s gestational limit. Clinic gestational age limits varied between clinics and were not stated in the paper. This is a larger proportion than the 24% of 18 to 24-year olds and 20% of 25-34-year olds who were turned away [44].

Under the sub-theme ‘Adolescent specific legislation’ key data related to ‘Parental notification law’ and ‘Judicial bypass’. In the US, parental notification and consent legislation varies from state to state [62]. Individual states require consent from at least one, sometimes both parents, others require only parental notification about the abortion procedure [62]. Two quantitative studies from the US investigated the effects of the introduction of parental notification laws on the ability of minors to obtain an abortion in New Hampshire [28] and Illinois [30]. Both studies found a decrease in adolescents seeking abortions following legislative change at 12 months [30] and two years [28].

Provider and frontline abortion clinic staff knowledge of adolescent-specific abortion legislation was high, greater than 90% of front-line staff interviewed [37] and greater than 50% of physicians interviewed [38]. Riley at al. found that physicians with adolescent children were supportive of the parental notification laws [31].

One study compared adolescent abortion across four different countries. It reported in two of the countries a different approach to the involvement of parents in adolescent abortion service seeking [54]. Swedish and Dutch health professionals supported adolescents to engage in discussions with their parents about their unintended pregnancy [54]. In Sweden, providers cannot legally talk to parents without adolescent consent [54].

Judicial bypass refers to an American bureaucratic process allowing adolescents to obtain an abortion without having to notify or receive parental consent by way of a judge granting permission. This was the focus of three quantitative studies [28, 37, 57] and two qualitative case studies [34, 48]. Judicial bypass is a costly and bureaucratic process [63]. The process involves judges questioning adolescents to determine if they are ‘mature’ enough to undergo an abortion. If their responses are deemed rational and intelligent, a judge may permit the adolescent to have an abortion [63]. If not, the adolescent has to carry the pregnancy to term [63]. Three studies identified ways in which adolescents received information about how to obtain a judicial bypass [28, 37, 57]. Adolescents did not receive correct information about their legal options. For example, one participant was incorrectly informed that abortions for adolescents under the age of 18 were not allowed in her state and another participant was not provided accurate information about the judicial bypass court process [57]. Participants in three studies state judicial bypass and parental notifications laws do not serve adolescents, instead address the beliefs of adults that adolescents lack rational and responsible decision-making capabilities [34, 48, 53]. Jacqueline, a participant from Coleman-Minahan et al. who was denied a judicial bypass, stated to her court-appointed guardian-ad-litem and the judge during her hearing,

*You guys keep telling me I’m not mature enough to make this decision and I don’t know what I’m getting myself into*, *yet*…*if I’m not mature enough to make a decision like this how am I mature enough to even have a baby and to go through the emotional and physical changes of having a kid*? [34]

However, in Deeb-Sossa et al., Estefania, an adolescent seeking an abortion, revealed how she was provided appropriate information and support about how to access abortion without her father having to be notified [48]. In Coleman-Minahan et al. the majority of adolescents stated that their attorneys and staff from the non-profit they had accessed provided them the most and at times only source of support. Jasmin, one participant, explained that her attorney “*cared more than just about the procedure*. *She actually asked me about my future and what I wanted to do…”* [34]. In the studies by Coleman-Minahan et al., Deeb-Sossa et al. and Ehrlich et al. adolescents described their experiences of being extensively questioned by judges and health professionals about their maturity and reasons for an abortion [34, 48, 57]. Adolescents expressed apprehension about travelling to court and the need for support in the court room [57]. Melissa, one of several participants, discussed being nervous, anxious about talking to the judge and fearful that she would not be granted a judicial bypass [57]. Coleman-Minahan et al. found in their study that adolescent’s experience of obtaining a judicial bypass were unpredictable, humiliating, caused emotional distress and for some was a traumatic experience [34].

### Components of abortion care

Three themes emerged within the domain that described the components of abortion care.

#### Confidentiality

Maintaining one’s confidentiality was an important aspect of abortion care. Concern around confidentiality was reported in three studies [46, 48, 57]. Ethnographic research with an adolescent, Estefania, found a desire for privacy when seeking abortion care was essential for her. Adolescents cited several reasons not to disclose their desire for an abortion to their parents. These reasons included: a belief that their parents would be upset, fear of adverse reactions, anticipated harm to their relationship with their parents, concern for their parent’s health, and a belief that they would be pressured to continue the pregnancy [46]. Adolescents expected difficult family relations, desired autonomy, and feared that news of their pregnancy and/or abortion would be shared with others [57].

#### Comprehensive care

‘Comprehensive care’ is a necessary component of abortion care. It encompasses the range of services and resources provided pre- and post-procedure. Ensuring individuals are provided and have access to all necessary information and care required [64]. The findings from six studies described ‘components of care’ which surround the actual abortion procedure, and included pre- and post-counselling, support and provision of contraception if desired. Three studies examined decision-making and the need for pregnancy options counselling [47, 53, 54] and three examined contraceptive counselling and follow-up with women at the point of care [35, 36, 55]. Experts interviewed in Welsh et al. stated that “*counselling was a crucial element of a clinic visit involving a teen’s request for pregnancy termination*” [54]. However, in other studies, adolescents reported that counselling was not necessary as they had already made their decision to have an abortion [47, 53]. For example in Brown’s 2013 study, that explored whether or not young British women would accept pregnancy options counselling, participants generally declined. Counselling was not necessary because they had already decided not to continue with the pregnancy [47].

Two studies explored the provision of contraception at the time of abortion as an aspect of comprehensive care. In Aiken et al. adolescent participants were significantly more likely (P< 0.001) than adults to obtain contraception from the non-profit abortion provider and choose an intrauterine contraceptive or implant [35]. In contrast adolescents in Israel, 67.6% were not provided a prescription for contraception peri-abortion [36].

#### Abortion procedure

Three studies focused on the experience and acceptability of the abortion procedure itself as part of ‘components of abortion care’. Phelps et al. studied the experiences of adolescents who had undergone complete, uncomplicated medical abortions [41]. The acceptability of the procedure appeared to increase over time [41]. Fielding et al. explored adolescents’ experience of medical abortion and their choices [49]. A 19-year-old participant stated that she chose medical over surgical abortion because it seemed to be less mentally distressing [49]. Another adolescent stated, “*cramping was the worst part*” [49] but was “*relieved that it is over with*” [49]. In contrast, in Halldén et al. adolescents found surgical more convenient and less confronting [51].

### Access to the health system

Five themes were included under this domain and are described below with associated sub-themes.

#### Delays in access

Studies found and examined delays that adolescents experienced at various points along their abortion care trajectory. These delays were either self-created (e.g., failing to recognise the unwanted pregnancy) or externally created (e.g., need for parental consent and judicial bypass). Studies also examined the effects that these delays experienced by adolescents had on their access to abortion care. The first sub-theme was ‘Individual delays in access’. In three studies, adolescents experienced a longer delay than adults in identifying that they were pregnant [21, 53, 58]. In Finer et al. <18-year olds took a week longer than all other age groups to suspect pregnancy [58]. Reasons for not suspecting pregnancy was examined in Mantovani et al. with asylum seekers in the UK under state care. Adolescents believed their symptoms were due to stress and changes in their environment [53].

The second sub-theme was ‘System-level delays’. In a New Zealand study, the age of woman had a statistically significant individual effect on the delay to abortion access. The average days between first visit with a referral doctor and the abortion for 20-year-olds and younger was 26.1 days. This delay gradually decreased as age increased [43]. The study by Finer et al. in the US found that on average ≤17-year-olds took more than 70 days from last menstrual period to the abortion. This was at least 10 days or more than adults [58].

Two studies from the US examined the effects of delays on adolescents access to abortion care. The studies found adolescents were more likely than older women to have an abortion later than 12 weeks of gestation [20, 45]. Eighteen to 19-year olds were significantly (P = 0.03) more likely than 20–24 year olds to have an abortion > 12 weeks [20], whilst 13 to 19 year olds were more likely (P = 0.04) than older woman (>19 years) [45].

#### Information about abortion procedures

Four studies examined information adolescents received concerning how to access abortion care, and the abortion procedure [33, 48, 49, 54]. Adolescent’s encountered abortion misinformation and inaccuracies in common social beliefs about the abortion procedure. This lead adolescents to avoid surgical abortion methods [49] and sought accurate information for themselves [33, 46]. In the Netherlands, information on how to access abortion care is provided to adolescents in school [54].

#### Decision-making process

Adolescents sought to make appropriate decisions for themselves. Adolescent’s decision-making was the beginning of their trajectory and access to the health system. In six studies, adolescents’ reasons for seeking an abortion were explored. These included their age, male partners/ boyfriends leaving, disruption of future life goals (education and career), current life circumstances, their inability to take care of and provide for the child, their relationship with their parents, pressure from parents or partner, and issues related to pregnancy, abortion, or adoption [29, 46, 47, 52, 57, 59]. Adolescents stated: *“…couldn't raise a child nor give it up for adoption”* [57], “…*pregnancy a mistake/accident”* [57], *“…I have a big future*. *He has a big future*. *It’s not something we want right now*. *It was a huge mistake”* [36], *“…abortion the best solution"*, *"couldn't go through with adoption”* [57], and *“because I felt that it’s unfair on the child if I bring it up now”* [52].

Four studies examined those with whom adolescents talk about making a decision. Adolescents appear to discuss their decision-making with influential adults (such as a clergyman, pastor, teachers, doctors, social workers) [53, 57], their mother/parents [42, 57], boyfriend or male partner [42, 53, 57], friends, or other family members. Some adolescents reported they did not talk to anyone as they had made the decision for themselves [47, 58] and realised the decision was their own [46]. One participant stated, “*Yeah*, *but I didn’t take it because I knew straight away as soon as I found out*. *I already knew that I didn’t want it*. *(R3*, *aged 17)”* [47].

Several factors were found to influence abortion decision-making, including expectations of negativity from adults around them [52, 53, 57], adolescents’ own cultural and/or religious beliefs [53], parent(s)’ reaction to their pregnancy [42, 51, 53, 57], and boyfriend/male partner leaving [53, 56]. Partner support was found to indicate less involvement of the mothers of adolescents [42]. Other factors influencing decision-making included male partner’s attitudes towards the pregnancy and/or abortion [42, 54], inability to financially support the child [46, 47, 51], and the effect on their future life plans [46, 51, 58]. For 24% of 15-to 19-year-olds in Chibber et al., their partner was the reason they sought an abortion [56].

#### Clinic operation and environment

Two sub-themes emerged under this theme. Under ‘Effects of protesters’, a study in the US found protesters appear to emotionally affect and upset adolescents [39].

The second sub-theme was ‘Telemedicine’. Telemedicine is an option for adolescents who are unable to travel long distances or cover the costs opted for when available [50]. Adolescent participants were comfortable with the idea as one 19-year-old stated “*Our generation*, *we’ve always done video chat and everything so it’s not awkward or anything…To me it kind of felt like the same thing [as an in-person visit with the doctor] just because I’ve grown up using computers”* [50]. Another adolescent felt telemedicine assisted her in accessing and talking to a doctor [50].

#### Structural barriers

Adolescents experience heightened ‘structural barriers’ along their care-seeking pathway accessing an abortion. These barriers were beyond the adolescents control and appear to be exacerbated by their age and context. They were part of the external context and environment, e.g., geography, transportation and payment. Adolescents faced transportation and financial challenges in accessing abortion [26, 40, 45, 46]. In the US, experts questioned in Welsh et al. stated that geographical location is a barrier for adolescents in accessing abortions, especially as rural areas lack abortion providers [54]. White et al. examined the association between travel distance and returning for the abortion procedure and found no association. However, of 18.8% of women who did not return for an abortion, 34.7% were <18 [32]. In Grindlay et al. study of telemedicine in the US, a 19-year-old participant discussed the difficulty of getting time off work and how the long-distance she would need to travel would compound the situation [50].

An 18-year-old participant in Fuentes et al. stated she lived more than a three-hour drive from the closest abortion clinic and was unsure if she would be able to cover the costs of travel or the abortion procedure [27]. Data from the US and Australia indicate adolescents had difficulties with transportation, experiencing delays, and difficulty paying [21, 26, 37, 40, 45, 46]. In Ely et al. adolescents experienced significantly higher (P<0.001) procedural costs and greater distances to travel than adult women in the US [26]. Experts from four high-income countries interviewed as part of the Welsh et al. reported in Sweden, the cost of an abortion is less than the contraceptive pill, while in the Netherlands, it is free [54].

## Discussion

This systematic review identified 35 studies in high-income countries which explored abortion care trajectories of adolescents. Directed content analysis identified themes which mapped to three domains of: context, components of care and access to services. Our analysis identified nine themes and eight sub-themes which elaborated the narrative around adolescent’s abortion care trajectory. We found that individual themes and subthemes interact with each other. Our study suggests that while adolescents face similar barriers to older women, when accessing an abortion service, they experience additional and unique legal barriers, concerns about confidentiality particularly in relation to parents, longer delays, and greater issues with costs and geographical distance. To the best of our knowledge this is the first systematic review looking at adolescents’ abortion trajectories.

### Abortion and the legal context

Our review identified that adolescent specific legislation does impact an adolescent’s personal care trajectory. Our search only identified studies from the US that examined adolescent experience of parental notification/consent laws; however, restrictive legislation for adolescents exists in several high-income countries. In Spain, parents or a legal guardian must be notified of a minors decision [65]. Adolescents under the age of 18 in Italy and Denmark require parental consent [66]. In contrast, in the Czech Republic, Iceland, Latvia and Portugal, parental consent is required for adolescents under the age of 16. There are other HICs, such as Sweden and Germany, where the law is not clear regarding parental consent. In Australia, parental consent and notification legislation concerning abortion vary across states [67]. We found that restrictive legislation in the US increased the likelihood of adolescent exposure to stigma, judgement and discrimination, stress, and trauma by infringing on adolescent’s rights to privacy, quality healthcare and information. While these findings might not be directly transferable to other countries and contexts, they suggest that enabling legislative frameworks must be considered in the provision of abortion care to adolescents.

### Comprehensive care

The review found that the provision of ‘comprehensive care’ was not consistent across studies. The research shows that adolescents were more likely to take up contraception when offered before and immediately after their abortion than when offered at follow-up appointments or at later points after an abortion. The review also found that the provision of abortion counselling to adolescents was carried out irrespective of whether the adolescents had already made their decision. Provision of abortion related information is regarded as quality practice [1], however, sensitivity training is needed to prevent unnecessary counselling. Tylee et al. [7], identified approaches to improve health provider’s abilities to provide adolescent-friendly services, including the use of guidelines, training, and quality-improvement strategies to improve provider performance. The findings from the review showed the need for consistent delivery of ‘comprehensive care’ to adolescents accessing abortion services, to ensure that adolescents are getting appropriate information, contraceptive and emotional counselling support.

### Access to abortion care–clinic operation

This reviewed found that adolescents experienced heightened barriers compared to adults, when trying to access abortion care. These barriers were due to distances travelled to services, cost (e.g., abortion procedure, transportation, and accommodation), interactions with protestors and loss of privacy. Our findings are consistent with existing research and *The Lancet Commission on Adolescent Health and Wellbeing* that show that adolescents face greater barriers and worse experience of care than adults when accessing health services for even non-stigmatized health issues [68, 69]. These barriers can further delay and complicate adolescent’s ability to access an abortion. The findings suggest that the provision of abortion via telemedicine may help to address these access barriers [1]. Adolescents are growing up immersed and at ease with the use of technology and the internet. It is not surprising that adolescents in our review stated that they would be satisfied with the delivery of medical abortion through telemedicine. Telemedicine can increase the number of abortions provided without stretching the workforce, as service providers can offer greater coverage from not being limited by geographical constraints [70]. Abortion via telemedicine is a safe abortion procedure with different service delivery models in Canada, Australia and the US [71]. Adolescent specific research on the use of telemedicine would be valuable to see how this service can address gaps and best meet needs of this population group.

### Limitations and strengths

This study was limited by the lack of studies in HICs that have examined adolescent access, navigation and experience of abortion care. As a result, decisions were made to include studies that were not directly focused on adolescents and were of variable quality. The heterogeneity of these studies did not allow for meta-analysis. We only included studies written in English and may have missed data from studies in other languages.

The strength of this review was that given the heterogeneity of study designs and samples, the directed content analysis using a framework helped make sense of diverse findings in order to answer the research question.

### Policy implications and recommendations

This review identified a gap in adolescent experience of access to care that are affected by stigma, and provider bias that affects the quality of care they receive. Dennis, Blanchard and Bessenaar have outlined indicators of quality abortion care service delivery [72]. These include quality guidelines, standards and indicators to ensure equity in the provision of abortion for adolescents. Standards of service provision are necessary to guide quality health care services that are likely to improve quality of services provided to adolescents [7, 73]. Young people in HICs frequently experience lower overall satisfaction than older adults [68]. Useful standards to assess services and improve them are the eight standards for quality of adolescent healthcare services outlined by Nair et al.: (1) adolescents’ health literacy; (2) community support; (3) appropriate package of services; (4) providers’ competencies; (5) facility characteristics; (6) equity and non-discrimination; (7) data and quality improvement; and (8) adolescents’ participation [74]. Improving provider’s performance will require more than the implementation of guidelines [73, 75]. Training of providers to deliver adolescent-friendly health services is also an important aspect of high-quality abortion delivery. The framework for adolescent-friendly health services is based on overcoming identified barriers that adolescents face when accessing a health service [7]. This is particularly important for abortion services, where adolescents may experience additional stigma related to judgement concerning their sexual activity and unintended pregnancy. An adolescent-friendly approach to providing care can assist in dealing with perceived and internalized stigma experienced by adolescents and biases of health professionals.

Adolescent voices are absent from the research. Highlighting the need for more participatory research involving adolescents propelling the research questions. Adolescents need to be involved in decision-making concerning their healthcare, and their values and preference for care incorporated into abortion care and models of care. Adolescents also need to be better engaged in the design of services to determine what can improve access and acceptability. This can be achieved through participatory approaches promoting the improvement and transformation of a service by addressing the collective needs of individuals at all levels, from managers to clients [76].

In some European countries’ adolescents are provided with information and support in school to access an abortion [77, 78]. This ‘adolescent centred’ comprehensive approach to sexual and reproductive education can be best provided within schools and communities. Adolescents should be involved in strategies to improve their health literacy [79] and healthcare providers must support adolescents to develop functional and critical health literacy skills as well [79].

## Conclusion

The majority of adolescent pregnancies in HICs are unintended. Adolescents face greater barriers than adults when accessing abortion care. Restrictive adolescent-specific abortion legislation and policy and the lack of affordable, non-judgemental and comprehensive services, knowledge of reproductive health and abortion services can result in delays in identifying and accessing care. There is also differences in the quality of abortion service delivery and the manner in which care is provided to adolescents that may not meet adolescent needs. This unmet need places adolescents at higher risk of STIs, unintended pregnancies, and unsafe abortions. Adolescents have the right to high-quality, non-judgemental, unbiased information and service provision in an adolescent-friendly environment. Removing legal barriers, improving health professional education, improved sex education and availability of telemedicine may enhance the access and quality of service provision, and adolescents overall experience of abortion care.

## Supporting information

S1 Table Search strategy. (DOCX)

S2 Table Quality assessment of included studies. (DOCX)

S3 Table Publication and study characteristics (n = 35). (DOCX)

S4 Table Prisma 2009 checklist. (DOC)
