# Supplementary material for: Abortion care pathways and service provision for adolescents in high-income countries: A qualitative synthesis of the evidence
Source: PLoS One. 2020 Nov 9;15(11):e0242015. doi: 10.1371/journal.pone.0242015 (PMC7652292; doi:10.1371/journal.pone.0242015)
Supplement: S1 Table — (DOCX) [file pone.0242015.s001.docx]

**S1 Table. Search terms**

| **Database** | **Search terms** |
| --- | --- |
| PubMed | (("pregnancy termination"[Title/Abstract]) OR ("termination of pregnancy"[Title/Abstract]) OR ("postconception fertility control"[Title/Abstract]) OR (abortion, legal[MeSH Terms]) OR ("menstrual regulation"[Title/Abstract]) OR (abortion*[Title/Abstract]) OR (abortion, induced[MeSH Terms])) NOT (("in vitro fertilization"[Title/Abstract]) NOT ("in vitro fertilisation"[Title/Abstract]) NOT (in vitro fertilization[MeSH Terms]) NOT ("threatened abortion"[Title/Abstract]) NOT (animals[MeSH Terms])) AND ((adolescent*[MeSH Terms]) OR (teenager*[Title/Abstract]) OR (teen*[Title/Abstract]) OR adolescent girl*[Title/Abstract])) AND ((health service*[MeSH Terms]) OR (adolescent health service*[MeSH Terms]) OR (reproductive health service*[MeSH Terms]) OR (abortion service*[Title/Abstract]) OR (youth friendly service*[Title/Abstract]) OR (adolescent friendly service[Title/Abstract]) OR ("service delivery" [Title/Abstract]) OR ("model of care"[Title/Abstract])) |
| Scopus | (TITLE-ABS-KEY(abortion*) OR TITLE-ABS-KEY("induced abortion*") OR TITLE-ABS-KEY("pregnancy termination") OR TITLE-ABS-KEY("termination of pregnancy") OR TITLE-ABS-KEY("menstrual regulation") OR TITLE-ABS-KEY(abortion, legal)) AND (TITLE-ABS-KEY(adolescent*) OR TITLE-ABS-KEY(teenager*) OR TITLE-ABS-KEY(teen*) OR TITLE-ABS-KEY(youth) OR TITLE-ABS-KEY("young people")) AND (TITLE-ABS-KEY("health service") OR TITLE-ABS-KEY("abortion service*") OR TITLE-ABS-KEY("adolescent friendly") OR TITLE-ABS-KEY("health pathways") OR TITLE-ABS-KEY("youth friendly")) |
| Popline | ((health service) OR (abortion service) OR (health pathway) OR (service pathway)) AND (adolescent OR teen OR teenager OR youth OR (young people)) AND ((induced abortion) OR abortion OR (termination of pregnancy) OR (pregnancy termination) OR (abortion, legal)) |
| Medline/CINAL | Adolescent   - AB adolescent* - AB teen* - AB teenager* - AB youth* - AB young people - AB "young people" - AB minor   S1 OR S2 OR S3 OR S4 OR S5 OR S6 OR S7  Abortion service   - AB "adolescent friendly service*" - AB "youth friendly service*" - (MH "Women's Health Services") OR (MH "Student Health Services") OR (MH "School Health Services") OR (MH "Community Health Services") OR (MH "Reproductive Health Services") - AB "abortion service*" - AB "reproductive health service*" - AB "adolescent health service*" - AB "service delivery" - AB "model of care"   S10 OR S11 OR S12 OR S13 OR S14 OR S15 OR S16 OR S17  Abortion   - (MH "Pregnancy, Unplanned") - AB "pregnancy termination" - AB "termination of pregnancy" - AB "postconception fertility control" - (MH "Abortion, Induced/ED/LJ/MT/ST/SN/TD/UT/CL") OR (MH "Abortion, Legal/AE/CL/ED/LJ/MT/TD/UT") - AB abortion* - AB "menstrual regulation" - AB "legal abortion" - AB "induced abortion"   S22 OR S23 OR S24 OR S25 OR S26 OR S27 OR S28 OR S29  Adolescent AND Abortion service AND Abortion |
| Embase | Abortion  abortion*.mp. or exp *legal abortion/ or exp *abortion/ or exp *induced abortion/  exp *pregnancy termination/ or termination of pregnancy.mp.  Adolescent  adolescent/  exp *adolescence/ or adolescent pregnancy/ or teen.mp.  juvenile/ or adolescent/  young people.mp. or young adult/  minor.mp. or "minor (person)"/  Abortion service  exp *health care delivery/ or abortion service.mp. or exp *health service/  exp sexual health/ or exp health care quality/ or exp health service/ or exp adolescent health/ or adolescent friendly service.mp.  sexual health/ or youth friendly service.mp.  health care quality/ or model of care.mp.  clinical pathway/ or health care delivery/ or health care policy/ or health service pathway.mp.  1 or 2  3 or 4 or 5 or 6 or 7  8 or 9 or 10 or 11 or 12  13 and 14 and 15 |
