# Supplementary material for: Abortion care pathways and service provision for adolescents in high-income countries: A qualitative synthesis of the evidence
Source: PLoS One. 2020 Nov 9;15(11):e0242015. doi: 10.1371/journal.pone.0242015 (PMC7652292; doi:10.1371/journal.pone.0242015)
Supplement: S2 Table — (DOCX) [file pone.0242015.s002.docx]

**S2 Table. Quality assessment of included studies (N=35)**

| **Quality assessment of cross-sectional studies using the Newcastle-Ottawa Scale (n=16)** | | | | | | | | | | | | | | | | | | | | | | | | | | | | | | | | | | |
| --- | --- | --- | --- | --- | --- | --- | --- | --- | --- | --- | --- | --- | --- | --- | --- | --- | --- | --- | --- | --- | --- | --- | --- | --- | --- | --- | --- | --- | --- | --- | --- | --- | --- | --- |
| **Reference** | **Country** | | | **Representativeness of the sample** | | | **Sample Size** | | | **Non-respondents** | | | | **Ascertainment of the exposure (risk factor)** | | | **The subjects in different outcome groups are comparable based on study design or analysis. Confounding factors are controlled** | | | | **Assessment of the outcome** | | | **Statistical test** | | | | | **Report number of outcome events or summary measures** | | | **Describe any efforts to address potential sources of bias** | | |
| Aiken et al. 2017[1] | England and Wales | | | - | | | + | | | - | | | | - | | | - | | | | - | | | - | | | | | - | | | + | | |
| Dodge, Haider, and Hacker 2012[2] | United States | | | + | | | + | | | ? | | | | - | | | - | | | | - | | | - | | | | | - | | | ? | | |
| Dodge, Haider, and Hacker 2013[3] | United States | | | + | | | + | | | - | | | | - | | | - | | | | - | | | - | | | | | - | | | ? | | |
| Ely et al. 2018[4] | United States | | | - | | | - | | | ? | | | | - | | | - | | | | - | | | - | | | | | - | | | + | | |
| Foster et al. 2013[5] | United States | | | - | | | + | | | ? | | | | - | | | - | | | | - | | | - | | | | | - | | | ? | | |
| Jones and Jerman 2017[6] | United States | | | - | | | + | | | - | | | | - | | | - | | | | - | | | - | | | | | - | | | + | | |
| Nickson, Smith, and Shelley 2006[7] | Australia | | | - | | | ? | | | - | | | | - | | | - | | | | - | | | - | | | | | - | | | ? | | |
| Pereira, Pires, and Canavarro 2019[8] | Portugal | | | - | | | - | | | ? | | | | - | | | - | | | | - | | | - | | | | | - | | | + | | |
| Phelps, Schaff, and Fielding 2001[9] | United States | | | + | | | + | | | - | | | | - | | | - | | | | - | | | - | | | | | - | | | ? | | |
| Preis, Rager, and Bershtling 2018[10] | Israel | | | - | | | - | | | ? | | | | - | | | - | | | | - | | | - | | | | | - | | | - | | |
| Ralph et al. 2014[11] | United States | | | + | | | + | | | - | | | | - | | | - | | | | - | | | - | | | | | - | | | + | | |
| Ramesh, Zimmerman, and Patel 2016[12] | United States | | | + | | | + | | | - | | | | - | | | - | | | | - | | | - | | | | | - | | | ? | | |
| Riley et al. 2015[13] | United States | | | + | | | + | | | - | | | | - | | | - | | | | - | | | - | | | | | - | | | ? | | |
| Silva, McNeill, and Ashton 2011[15] | New Zealand | | | - | | | + | | | - | | | | - | | | - | | | | - | | | - | | | | | - | | | - | | |
| Upadhyay et al. 2014[16] | United States | | | - | | | + | | | ? | | | | - | | | - | | | | - | | | - | | | | | - | | | ? | | |
| Zapka et al. 2001[17] | United States | | | + | | | + | | | - | | | | - | | | - | | | | - | | | - | | | | | - | | | - | | |
| **Quality assessment of cohort studies using Newcastle-Ottawa Scale (n=2)** | | | | | | | | | | | | | | | | | | | | | | | | | | | | | | | | | | |
| **Reference** | **Country** | | | **Representativeness of the exposed cohort** | | **Selection of the non-exposed cohort** | | | **Ascertainment of exposure** | | | **Demonstration that outcome of interest was not present at start of study** | | | | **Comparability of cohorts on the basis of the design or analysis** | | | **Assessment of the outcome** | | | **Was follow-up long enough for outcomes of occur** | | | | **Adequacy of follow-up of cohorts** | | | | **Report number of outcome events or summary measures** | | | **Describe any efforts to address potential sources of bias** | |
| MacAfee, Castle, and Theiller 2015[18] | United States | | | - | | + | | | - | | | - | | | | - | | | - | | | - | | | | ? | | | | - | | | ? | |
| White, Turan, and Grossman 2017[19] | United States | | | + | | + | | | - | | | - | | | | - | | | - | | | - | | | | - | | | | - | | | + | |
| **Quality assessment of qualitative studies using Critical Appraisal Skills Programme checklist (n=13)** | | | | | | | | | | | | | | | | | | | | | | | | | | | | | | | | | | |
| **Reference** | **Country** | | | **Research aims** | | **Methodology** | | | **Research design** | | | **Recruitment strategy** | | | | **Data collection** | | | **Data analysis** | | | **Reflectivity** | | | | **Ethical consideration** | | | | **Findings** | | | **Value of research** | |
| Andrews and Boyle 2003[20] | United States | | | - | | - | | | - | | | - | | | | - | | | + | | | + | | | | - | | | | - | | | - | |
| Brown 2013[21] | England | | | - | | - | | | - | | | ? | | | | - | | | ? | | | - | | | | - | | | | + | | | ? | |
| Coleman-Minahan et al. 2018[22] | United States | | | - | | - | | | - | | | - | | | | - | | | + | | | - | | | | - | | | | - | | | - | |
| Deeb-Sossa and Billings 2014[23] | United States | | | - | | - | | | - | | | - | | | | - | | | + | | | + | | | | + | | | | - | | | - | |
| Falk, Brynhildsen, and Ivarsson 2009[24] | Sweden | | | - | | - | | | - | | | - | | | | - | | | - | | | - | | | | - | | | | - | | | - | |
| Fielding, Edmunds, and Schaff 2002[25] | United States | | | + | | - | | | - | | | - | | | | - | | | + | | | + | | | | - | | | | - | | | - | |
| Fuentes et al. 2016[26] | United States | | | - | | - | | | - | | | - | | | | - | | | + | | | - | | | | - | | | | - | | | - | |
| Grindlay, Lane, and Grossman 2013[27] | United States | | | - | | + | | | - | | | - | | | | - | | | + | | | + | | | | + | | | | - | | | - | |
| Halldén, Christensson, and Olsson 2005[28] | Sweden | | | - | | - | | | - | | | - | | | | - | | | + | | | - | | | | - | | | | - | | | - | |
| Kirkman et al. 2011[29] | Australia | | | - | | - | | | - | | | - | | | | - | | | + | | | - | | | | - | | | | - | | | - | |
| Mantovani and Thomas 2014[30] | United States | | | - | | + | | | ? | | | - | | | | - | | | + | | | - | | | | - | | | | - | | | - | |
| O’Donnell et al. 2018[31] |  | | | - | | - | | | - | | | - | | | | - | | | + | | | - | | | | - | | | | - | | | - | |
| Welsh, McCarthy, and Cromer 2001[32] | United States, United Kingdom, Sweden and Netherlands | | | + | | - | | | - | | | - | | | | - | | | ? | | | - | | | | - | | | | - | | | ? | |
| **Quality assessment of mixed method studies using the Mixed Methods Appraisal Tool (n=4)** | | | | | | | | | | | | | | | | | | | | | | | | | | | | | | | | | | |
| **Reference** | | **Country** | Screening questions | | | | | Qualitative | | | | | | | | | | Quantitative | | | | | | | | | Mixed method | | | | | | | |
|  |  |  | **Are there clear qualitative and quantitative research questions (or objectives)** | | **Do the collected data allow address the research question (objective)?** | | | **Are the sources of qualitative data relevant to address the research question (objective)?** | | | **Is the process for analysing qualitative data relevant to address the research question (objective)?** | | **Is appropriate consideration given to how findings relate to the context?** | | **Is appropriate consideration given to how findings relate to researchers' influence?** | | | **Is the sampling strategy relevant to address the quantitative research question?** | | **Is the sample representative of the population understudy?** | | | **Are measurements appropriate?** | | **Is there an acceptable response rate (60% or above)?** | | | **Is the mixed methods research design relevant to address the qualitative and quantitative research questions (or objectives), or the qualitative and quantitative aspects of the mixed methods question (or objective)?** | | | **Is the integration of qualitative and quantitative data (or results) relevant to address the research question (objective)?** | | | **Is appropriate consideration given to the limitations associated with this integration?** |
| Chibber et al. 2014[33] | | United States | + | | - | | | - | | | - | | - | | + | | | - | | + | | | ? | | ? | | | - | | | - | | | + |
| Ehrlich 2003[34] | | United States | + | | - | | | - | | | - | | - | | + | | | - | | + | | | ? | | + | | | - | | | - | | | + |
| Finer et al. 2005[35] | | United States | + | | - | | | - | | | - | | - | | + | | | - | | - | | | - | | - | | | - | | | - | | | ? |
| Finer et al. 2006[36] | | United States | + | | - | | | - | | | - | | - | | + | | | - | | - | | | - | | ? | | | - | | | - | | | ? |

*Note: (-) low risk of bias; (+) high risk of bias, (?) unclear*

**References**

1. Aiken A, Lohr P, Aiken C, Forsyth T, Trussell J. Contraceptive method preferences and provision after termination of pregnancy: a population-based analysis of women obtaining care with the British Pregnancy Advisory Service. BJOG. 2017;124:815-24. doi: <http://dx.doi.org/10.1111/1471-0528.14413>.

2. Dodge LE, Haider S, Hacker MR. Knowledge of state-level abortion laws and policies among front-line staff at facilities providing abortion services. Women's Health Issues: Official Publication Of The Jacobs Institute Of Women's Health. 2012;22(5):e415-e20. doi: 10.1016/j.whi.2012.07.002. PubMed PMID: 22944899.

3. Dodge LE, Haider S, Hacker MR. Knowledge of state-level abortion laws and regulations among reproductive health care providers. Womens Health Issues. 2013;23(5):e281-6. Epub 2013/08/06. doi: 10.1016/j.whi.2013.06.003. PubMed PMID: 23910426.

4. Ely GE, Hales TW, Jackson DL, Kotting J, Agbemenu K. Access to choice: examining differences between adolescent and adult abortion fund service recipients. Health and Social Care in the Community. 2018;26:695-704.

5. Foster DG, Kimport K, Gould H, Roberts SC, Weitz TA. Effect of abortion protesters on women's emotional response to abortion. Contraception. 2013;87(1):81-7.

6. Jones RK, Jerman J. Characteristics and circumstances of U.S. Women who obtain very early and second-trimester abortions. PloS one. 2017;12 (1) (no pagination)(e0169969). doi: <http://dx.doi.org/10.1371/journal.pone.0169969>.

7. Nickson C, Smith AMA, Shelley JM. Travel undertaken by women accessing private Victorian pregnancy termination services. Aust New Zealand J Public Health. 2006;30(4):329-33. doi: 10.1111/j.1467-842X.2006.tb00844.x.

8. Pereira J, Pires R, Canavarro MC. Decision-making trajectories leading to termination of an unplanned pregnancy: specificities among adolescent and adult women. Journal of Reproductive and Infant Psychology. 2019;37(3):242-55.

9. Phelps RH, Schaff EA, Fielding SL. Mifepristone abortion in minors. Contraception. 2001;64(6):339-43. PubMed PMID: 11834231.

10. Preis H, Prager M, Bershtling O. Abortion among adolescents in Israel: Intervention and health behavior outcomes. Health & Social Work. 2018;43(4). doi: doi: 10.1093/hsw/hly022.

11. Ralph L, Gould H, Baker A, Foster DG. The Role of Parents and Partners in Minors' Decisions to Have an Abortion and Anticipated Coping After Abortion. J Adolesc Health. 2014;54(4):428-34. doi: 10.1016/j.jadohealth.2013.09.021. PubMed PMID: 104049909. Language: English. Entry Date: 20140331. Revision Date: 20150710. Publication Type: Journal Article.

12. Ramesh S, Zimmerman L, Patel A. Impact of Parental Notification on Illinois Minors Seeking Abortion. The Journal Of Adolescent Health: Official Publication Of The Society For Adolescent Medicine. 2016;58(3):290-4. doi: 10.1016/j.jadohealth.2015.11.004. PubMed PMID: 26794433.

13. Riley M, Ahmed S, Reed BD, Quint EH. Physician Knowledge and Attitudes around Confidential Care for Minor Patients. Journal of Pediatric and Adolescent Gynecology. 2015;28(4):234-9. doi: 10.1016/j.jpag.2014.08.008.

14. Rose SB, Garrett SM, Stanley J. Immediate postabortion initiation of levonorgestrel implants reduces the incidence of births and abortions at 2 years and beyond. Contraception. 2015;92(1):17-25. doi: 10.1016/j.contraception.2015.03.012.

15. Silva M, McNeill R, Ashton T. Factors affecting delays in first trimester pregnancy termination services in New Zealand. Aust New Zealand J Public Health. 2011;35(2):140-5. doi: 10.1111/j.1753-6405.2010.00659.x.

16. Upadhyay UD, Weitz TA, Jones RK, Barar RE, Foster DG. Denial of abortion because of provider gestational age limits in the United States. Am J Public Health. 2014;104(9):1687-94. doi: 10.2105/AJPH.2013.301378.

17. Zapka JG, Lemon S, Peterson LE, Palmer H, Goldman MB. The silent consumer: women’s reports and ratings of abortion services. Medical Care. 2001;39(1):50-60.

18. MacAfee L, Castle J, Theiler RN. Association between the New Hampshire parental notification law and minors undergoing abortions in Northern New England. Obstet Gynecol. 2015;125(1):170-4. doi: 10.1097/AOG.0000000000000585.

19. White K, Turan JM, Grossman D. Travel for abortion services in Alabama and delays obtaining care. Women's Health Issues. 2017;27(5):523-9. doi: <http://dx.doi.org/10.1016/j.whi.2017.04.002>.

20. Andrews J, Boyle J. African American adolescents' experiences with unplanned pregnancy and elective abortion. Health Care for Women International. 2003;24(5):414-33. doi: <https://doi.org/10.1080/07399330390212199>.

21. Brown S. Is counselling necessary? Making the decision to have an abortion. A qualitative interview study. European Journal of Contraception and Reproductive Health Care. 2013;18(1):44-8. doi: 10.3109/13625187.2012.750290.

22. Coleman-Minahan K, Stevenson AJ, Obront E, Hays S. Young Women’s Experiences Obtaining Judicial Bypass for Abortion in Texas. J Adolesc Health. 2018:1-6. doi: <https://doi.org/10.1016/j.jadohealth.2018.07.017>.

23. Deeb-Sossa N, Billings DL. Barriers to abortion facing Mexican immigrants in North Carolina: Choosing folk healers versus standard medical options. Latino Studies. 2014;12(3):399-423. PubMed PMID: 33882405.

24. Falk G, Brynhildsen J, Ivarsson AB. Contraceptive counselling to teenagers at abortion visits--a qualitative content analysis. The European Journal Of Contraception & Reproductive Health Care: The Official Journal Of The European Society Of Contraception. 2009;14(5):357-64. doi: 10.3109/13625180903171815. PubMed PMID: 19916762.

25. Fielding SL, Edmunds E, Schaff EA. Having an abortion using mifepristone and home misoprostol: a qualitative analysis of women’s experiences. Perspectives on Sexual and Reproductive Health. 2002;34(1):34-40.

26. Fuentes L, Lebenkoff S, White K, Gerdts C, Hopkins K, Potter JE, et al. Women's experiences seeking abortion care shortly after the closure of clinics due to a restrictive laws in Texas. Contraception. 2016;93(4):292-7. doi: 10.1016/j.contraception.2015.12.017.

27. Grindlay K, Lane K, Grossman D. Women's and providers' experiences with medical abortion provided through telemedicine: a qualitative study. Women's Health Issues. 2013;23(2):e117-e22. doi: <http://dx.doi.org/10.1016/j.whi.2012.12.002>.

28. Halldén B-M, Christensson K, Olsson P. Meanings of Being Pregnant and Having Decided on Abortion: Young Swedish Women's Experiences. Health Care for Women International. 2005;26(9):788-806. doi: <https://doi.org/10.1080/07399330500230961>.

29. Kirkman M, Rowe H, Hardiman A, Rosenthal D. Abortion is a difficult solution to a problem: A discursive analysis of interviews with women considering or undergoing abortion in Australia. Women's Studies International Forum. 2011;34:121 - 9. doi: <http://dx.doi.org/10.1016/j.wsif.2010.11.002>.

30. Mantovani N, Thomas H. Choosing motherhood: The complexities of pregnancy decision-making among young black women 'looked after' by the State. Midwifery. 2014;30(3):72-8. doi: 10.1016/j.midw.2013.10.015.

31. O’Donnell J, Goldberg A, Lieberman E, Betancourt T. “I wouldn’t even know where to start”: unwanted pregnancy and abortion decision-making in Central Appalachia. Reproductive Health Matters. 2018;26(54):98-112. doi: DOI:10.1080/09688080.2018.1513270.

32. Welsh P, McCarthy M, Cromer B. Abortion in adolescence: A four-country comparison. Women's Health Issues. 2001;11(2):73-9. doi: 10.1016/S1049-3867(00)00078-5.

33. Chibber KS, Biggs MA, Roberts SC, Foster DG. The Role of Intimate Partners in Women’s Reasons for Seeking Abortion. Women's Health Issues. 2014;24(1). doi: <http://dx.doi.org/10.1016/j.whi.2013.10.007>.

34. Ehrlich JS. Grounded in the reality of their lives: listening to teens who make the abortion decision without involving their parents. Berkeley Womens Law Journal. 2003;18(1):61-180. Epub 2004/05/26. doi: <http://dx.doi.org/https://doi.org/10.15779/Z38D21RH72>. PubMed PMID: 15156878.

35. Finer LB, Frohwirth LF, Dauphinee LA, Singh S, Moore AM. Reasons U.S. women have abortions: Quantitative and qualitative perspectives. Perspectives on Sexual and Reproductive Health. 2005;37(3):110-8. doi: 10.1363/3711005.

36. Finer LB, Frohwirth LF, Dauphinee LA, Singh S, Moore AM. Timing of steps and reasons for delays in obtaining abortions in the United States. Contraception. 2006;74(4):334-44. doi: 10.1016/j.contraception.2006.04.010.
