# Supplementary material for: Abortion care pathways and service provision for adolescents in high-income countries: A qualitative synthesis of the evidence
Source: PLoS One. 2020 Nov 9;15(11):e0242015. doi: 10.1371/journal.pone.0242015 (PMC7652292; doi:10.1371/journal.pone.0242015)
Supplement: S3 Table — (DOCX) [file pone.0242015.s003.docx]

**Supplementary table S3. Publication and study characteristics (n=35)**

| **Reference** | **Country** | **Study period** | **Setting** | **Sample** | **Aim** | **Design** |
| --- | --- | --- | --- | --- | --- | --- |
| Aiken et al. 2017[22] | England and Wales | 1 January 2011 to 31 December 2014 | British Pregnancy Advisory Service (BPAS) | Women obtaining TOP and offered contraceptive counselling at BPAS. | The objectives were to: (1) examine women’s chosen methods of contraception, including by type of TOP and pathway to method provision; and (2) assess the fulfilment of contraceptive choices post-TOP. | Quantitative cross-sectional survey. |
| Andrews and Boyle 2003[46] | United States | Not stated | Non-profit clinic providing women’s health services in a large south-eastern city. | 12 African American females aged 15 to 18 years of age in the process of obtaining an elective abortion and having the ability to recall and verbally convey information with reasonable clarity and accuracy. | To generate an interpretive theory about how African American adolescents, experience unplanned pregnancy and elective abortion. | Qualitative prospective ethnographic study.  Using semi-structured one-on-one interviews. |
| Brown 2013[47] | United Kingdom | Not stated | Recruited from day-patients on a surgical termination of pregnancy list at a Women and Children’s Unit in the north of England. | 24 women aged between 16 and 20 who were waiting for, or had recently had, a surgical abortion. | The purpose of the study was to explore young women’s knowledge of contraception, and attitudes towards and decisions about contraceptive use and abortion. | Qualitative cross-sectional study.  Using semi-structured one-on-one interviews. |
| Chibber et al. 2014[23] | United States | 2008 and 2010 | 30 abortion care facilities | English or Spanish speaking, Women 15 years or over who are either just over the facility’s gestational age limit or denied an abortion (n = 231), or women just under the limit and who received an abortion (n = 452), or women receiving a first trimester procedure (n=273). | The purpose of the study is to examine how partners figure in women’s abortion decisions, and identifies factors associated with identifying partner as a reason (PAR) for abortion. | Baseline data from prospective mixed method longitudinal study (Turnaway Study).  Using a survey. |
| Coleman-Minahan et al. 2018[38] | United States | September to December 2016 | Recruited from client records at Jane’s Due Process, a non-profit organisation in Texas providing legal representation. | 20 adolescents 16 to 19 years of age who had tried to obtain a judicial bypass to obtain an abortion without parental consent. | The purpose is to draw on qualitative interviews to explore adolescent’s experiences with the judicial bypass process in Texas. | Qualitative retrospective study.  Using telephone interviews. |
| Deeb-Sossa and Billings 2014[24] | United States | Not stated | Not-for-profit community health clinic “Care Inc.” in North Carolina. | A Mexican immigrant teen, women, and service providers. | The purpose is to analyse how Mexican immigrant women and teens living in North Carolina negotiate numerous legal institutional barriers when seeking abortions. | Qualitative ethnographic study.  Using one-on-one interviews. |
| Dodge, Haider, and Hacker 2012[39] | United States | Not stated | Most restrictive and least restrictive states based on their abortion laws and policies. | 46 front-line staff members at facilities providing abortion care in 11 states in the United States. States were not identified. | The primary aim of this study was to assess the accuracy of the information regarding state-level abortion laws and policies received by a simulated patient from front-line staff members at facilities providing abortion services. | Quantitative cross-sectional simulation-based study.  Using telephone survey. |
| Dodge, Haider, and Hacker 2013[40] | United States | June 2010 to April 2011. | Recruited in person from the 2010 American Congress of Obstetrics and Gynaecology Annual Clinical Meeting and online through emails sent to member lists of four reproductive health professional organizations. | 282 clinicians, members of several reproductive health professional organizations. | The purpose of the study is to assess knowledge of state-level abortion laws and regulations among clinicians who provide reproductive health care. | Quantitative cross-sectional online survey.  Using online survey. |
| Ehrlich 2003[54] | United States | May 1998 - April 1999 quantitative interviews  June 1998 – November 1999 qualitative interviews | Recruited through Planned Parenthood League of Massachusetts. | 490 individuals’ clinical records.  26 in-depth interviews with minors who had received judicial authorization for an abortion. | The purpose of the study is looking at how young women made the abortion decision, what their reasons were for not involving their parents, whom they involved, and, lastly, what It was like for them to seek court authorization. | Mixed method cross-sectional study.  Using one-on-one in-depth interviews. |
| Ely et al. 2018[25] | United States | 2010 to 2015 | National Network of Abortion Funds (NNAF) Tiller Memorial Fund | 3,288 women receiving financial assistance from the National Network of Abortion Fund’s.  481 aged 17 or younger  2807 aged 18 to 49 years of age | The purpose was to compare the experiences of adolescent abortion fund recipients to those of adults and discuss the results of this comparison from a trauma-informed perspective. | Quantitative cross-sectional study. |
| Falk, Brynhildsen, and Ivarsson 2009[48] | Sweden | Starting in January 2006. |  | 36 clinical records were consecutively selected. | The aim of the study was to examine the notes in the medical records pertaining to the visits of teenagers requesting an abortion. Assessing how earlier contraceptive use was documented and how future contraception was planned as a reflection of what was discussed between the teenager and the caregiver. | Qualitative retrospective study.  Using content analysis of clinical record data. |
| Fielding, Edmunds, and Schaff 2002[49] | United States | Not stated | Clinic affiliated with Reproductive Health Program at the University of Rochester. | 43 women, part of a clinical trial of abortion using mifepristone. | The purpose of this study was to develop findings to provide clinicians with a more detailed understanding of how patients view induced abortion using mifepristone. | Qualitative cross-sectional study.  Using f open-ended questionnaire and one-on-one in-depth interviews. |
| Finer et al. 2005[56] | United States | From December 2003 to March 2004. | 11 large abortion providers, one from each of the nine major United States geographic regions. | 1209 women arriving at abortion facilities for a termination completed the questionnaire.  242 women aged 19 years or younger  38 English-speaking women obtaining abortions or having an abortion follow-up visits at four clinics were recruited for the in-depth interview.  9 women aged 19 years or younger | To understand women’s reasons for having abortions. | Mixed method cross-sectional study.  Using self-administered questionnaires and one-on-one in-depth interviews in English and Spanish. |
| Finer et al. 2006[55] | United States | From December 2003 to March 2004. | 11 large abortion providers, one from each of the nine major United States geographic regions. | 1209 women arriving at abortion facilities for a termination completed the questionnaire.  242 women aged 19 years or younger.  38 English-speaking women obtaining abortions or having an abortion follow-up visits at four clinics were recruited for the in-depth interview.  9 women aged 19 years or younger. | To examine the steps in the process of obtaining abortions and women's reported delays to help understand difficulties in accessing abortion services. | Mixed method cross-sectional study.  Using self-administered questionnaires and one-on-one in-depth interviews in English and Spanish. |
| Foster et al. 2013[41] | United States | 2008 and 2010 | 30 abortion care facilities and informants from 27 of these facilities. | 956 women completed baseline interview (273 = first trimester; 452 = near-limit abortion group; 231 = Turnaways.  173 women aged 14 to 17 years. | To seek to understand who experiences and is upset by protesters and how protesters affect women's emotional responses to an abortion. | Quantitative prospective longitudinal study (Turnaway Study).  Using one-on-one interviews. |
| Fuentes et al. 2016[26] | United States | November 2013 – June 2014 and October – November 2014 | Texas clinics no longer providing abortion care | 23 English and Spanish speaking women aged 18 years and over. 2 18-year-olds included. | Aim to describe women’s experiences seeking abortion care shortly after clinics closed and document pregnancy outcomes of women affected by these closures. | Qualitative cross-sectional study.  Using one-on-one semi-structured interviews. |
| Grindlay, Lane, and Grossman 2013[50] | United States | October 2009 – February 2010 | Planned Parenthood of the Heartland clinics in Iowa. | Women at least 18 years of age, able to speak English, at least 63 days gestation.  20 women receiving telemedicine, 5 women receiving in-person provision.  15 clinic staff involved with standard method of providing medical abortion and those involved with the telemedicine model. | The purpose was to evaluate women’s and providers’ experiences with telemedicine provision of medical abortion qualitatively. Aimed to learn more about the acceptability of the telemedicine abortion service and the impact that it has on patients, staff, and clinic operations. | Qualitative cross-sectional study.  Using one-on-one in-depth interviews. |
| Halldén, Christensson, and Olsson 2005[51] | Sweden | First half 2003 | A youth clinic or two gynaecology outpatients’ clinics in one midsize and one large city. | 10 18 to 20 year of women wo spoke Swedish. | To illuminate meanings of the phenomenon of “being pregnant and having decided on abortion”. | Qualitative cross-sectional study.  Using reflective one-on-one interviews. |
| Jones and Jerman 2017[17] | United States | 2014 | National sample from 87 non-hospital facilities across the country. | 947 women < 15 to 19-year-old women (study sample <15 to 35+). | To determine which characteristics and circumstances were associated with obtaining very early and second-trimester abortions. | Quantitative cross-sectional study.  Using self-administered questionnaire. |
| Kirkman et al. 2011[52] | Australia | February 2007 to February 2008 | Pregnancy Advisory Service in Melbourne, Australia | 136 women either aged 16 to 18 or living in rural or regional areas, 12-18 weeks gestation.  50 women aged 16 to 18 years of age. | The purpose was to understand what it means to women in the state of Victoria, Australia, to have a pregnancy during which they contemplate or undergo an abortion. | Qualitative cross-sectional study.  Using telephone interviews. |
| MacAfee, Castle, and Theiler 2015[27] | United States | January 2011 to December 2012 | Planned Parenthood of Northern New England clinic sites | 373 women younger than 18 years of age who had a medical or surgical abortion at any of the Planned Parenthood. | The primary objective of this study was to determine whether the parental notification law resulted in a change in the number of minors seeking abortions at Planned Parenthood clinics in New Hampshire, Vermont, and Maine.  The secondary objective included evaluating changes in the age and gestational age of minors seeking abortions and assessing rates of parental involvement among northern New England minors seeking abortions. | Quantitative retrospective cohort study.  Using clinical records. |
| Mantovani and Thomas 2014[28] | United Kingdom | 2005 to 2007 | London Local Authorities selected for their geographical diversity. | 15 young women aged 16 to 19, from black minority ethnic groups with a history of care. | The study seeks to address two research questions: how interpersonal relationships affect the decision-making-process of an unexpected pregnancy among informants, and what is there experience of health professionals during decision-making? | Qualitative cross-sectional study.  Using one-on-one interviews conducted in participants’ homes. |
| Nickson, Smith, and Shelley 2006[42] | Australia | November 2002 to June 2003 | 5 out of 8 private pregnancy termination service providers in metropolitan Melbourne. | 1,244 women, Australian residents living in Victoria, accessing private abortion care in metropolitan Melbourne.  149 women aged 15 to 19 years of age | The purpose is to investigate the extent and cost of travel undertaken by women accessing Victorian termination of pregnancy services. | Quantitative cross-sectional observational study.  Using self-administered questionnaire. |
| O’Donnell et al. 2018[37] | United States | December 2013 to September 2014 | Three specialised reproductive healthcare facilities offering abortion care; two federally funded primary health care centres; and two centres of commerce in Kentucky, Tennessee, and Virginia.  Central Appalachia | 31 English-speaking women aged 16 to 45 years, residing in Central Appalachia.  4 women aged 16 to 20 years of age. | The purpose was to determine what barriers do women of reproductive age who live in rural counties in Central Appalachia face when seeking reproductive health care and what facilities access to care? | Qualitative retrospective study.  Using semi-structured interviews. |
| Pereira, Pires, and Canavarro 2019[30] | Portugal | September 2013 and August 2016 | 16 healthcare services that provide abortion. | 422 women who terminated an unplanned pregnancy.  248 adolescents  174 adults | The purpose to describe the decision-making trajectories leading to termination of an unplanned pregnancy and to explore the differences according to women’s age. | Quantitative cross-sectional study. |
| Phelps, Schaff, and Fielding 2001[43] | United States | May 1998 to October 2000 | Rochester, New York | 28 adolescents 14- to 17-years-old, 56 days’ gestation age, and seeking abortion care with parental consent from at least one parent. | The purpose was to determine whether medical abortion with mifepristone and misoprostol is acceptable and well tolerated in a sample of 14 to 17-year-old adolescents. | Quantitative prospective pilot study  Using a questionnaire. |
| Preis, Prager, and Bershtling 2018[29] | Israel | February 2015 to March 2016 | 10 hospitals spread through Israel and covered large geographical areas. | 172 adolescents under the age of 19 who underwent an abortion. | The purpose was to examine the post-abortion reproductive health behaviours of adolescents under the age of 19 and pre-abortion interventions that may have influenced them. | Quantitative prospective study. |
| Ralph et al. 2014[31] | United States | 2008 | Private abortion clinic | 476 women 17 years and younger | The purpose was to explore the extent and nature of parental and partner involvement in minors' abortion decision making and its influence on minors' confidence in and predicted ability to cope with their decision. | Quantitative descriptive analysis of clinical records.  Using clinical records and counselling needs assessment forms. |
| Ramesh, Zimmerman, and Patel 2016[32] | United States | August 15, 2012 to August 14, 2013 (prior) and August 15, 2013 to August 1, 2014 (after) the Illinois Parent Notification Act was put into effect. | Reproductive Health Services clinic at John H. Stroger Jr Hospital of Cook County.  Public urban hospital in Chicago. | 12 to 17-year-olds obtaining a first-trimester abortion  18 -to 21-year-olds unaffected by the law was the control. | The aim was to describe the impact of the Illinois Parental Notification of Abortion Act on minors presenting for first-trimester abortion at an urban clinic in Chicago, Illinois. | Quantitative retrospective pre-post-test study. |
| Riley et al. 2015[33] | United States | 2013 | Department of Family Medicine, Medicine-paediatrics, Obstetrics and Gynaecology and Paediatrics at the University of Michigan. | 259 faculty, fellows and resident physicians. | The aim was to assess physicians’ knowledge of these laws, attitudes around the provision of confidential care to minors and barriers to providing confidential care. | Quantitative cross-sectional study.  Using online survey. |
| Silva, McNeill, and Ashton 2011[44] | New Zealand | Februarys to May 2009. | Nine first trimester abortion clinics in the country. Eight clinics were on the North Island, one in the South Island. | 2,950 women attending participating abortion clinics records were audited.  Of these women 1,086 women completed a questionnaire. | The aim was to identify the factors affecting the timeliness of services in first trimester abortion services in New Zealand. | Quantitative multi-method cross-sectional study.  Using clinical records and self-administered questionnaires. |
| Upadhyay et al. 2014[36] | United States | 2008 to 2010 | 30 abortion facilities across 21 states. | 956 women completed baseline interview (273 = first trimester; 452 = near-limit abortion group; 231 = Turnaways.  173 women aged 14 to 17 years. | The purpose was to examine the factors influencing delay in seeking abortion and the outcomes for women denied abortion care because of gestational age limits at abortion facilities. | Baseline data from prospective mixed method longitudinal study (Turnaway Study).  Using a survey. |
| Welsh, McCarthy, and Cromer 2001[53] | Sweden, Netherlands, Britain, and United States | Not stated | Not stated | Professionals from either a personal or public health perspective had expertise in the field of adolescent pregnancy (n=75).  The Netherlands = 18, US = 18, Great Britain = 19, Sweden = 20. | The purpose of the study was to conduct a comparison of perceptions concerning abortion among health care providers, administrators, politicians and anti-abortion activities. | Qualitative  Using semi-structured one-on-one interviews. |
| White, Turan, and Grossman 2017[35] | United States | 2013 | Two Alabama abortion clinics. | 2,730 women attended initial consultation  121 women aged less than 18 years of age. | The purpose of this study is to assess the association between one-way distance travelled for abortion services and women’s return for the procedure after the mandatory consultation and waiting period in Alabama. | Quantitative retrospective cohort study.  Using de-identified billing data. |
| Zapka et al. 2001[45] | United States | July 1996 to October 1997 | Two health centres. Vermont non-profit private group practice, most surgical abortions performed by female physician.  New Hampshire women’s health centre, 1 male physician performs surgical abortions. | 797 women who had an outpatient first or second trimester surgical abortion. 171 participants were women aged 13 to 19 years of age. | The purpose of this study is to assess women’s experiences with abortion services and to gather information on the dimensions of access and satisfaction to guide quality improvement efforts. | Quantitative cross-sectional study Using clinical records and surveys. |
